# Flow-controlled expiration discloses PEEP-dependent dynamic hysteresis of the pressure-volume loop

**DOI:** 10.1186/cc10712

**Published:** 2012-03-20

**Authors:** S Schumann, L Vimlati, M Lichtwarck-Aschoff, J Guttmann

**Affiliations:** 1 University Medical Center Freiburg, Germany; 2 Uppsala University, Uppsala, Sweden

## Introduction

Hysteresis of the pressure-volume loop is a measure for the additional energy that is required during inspiration to recruit and inflate additional alveoli. The hysteresis area is usually constructed using data from a low-flow inflation/deflation maneuver; that is, from a quasi-static situation that the lung never sees during ongoing ventilation. However, during the dynamic conditions of mechanical ventilation the hysteresis area is biased by resistive pressure portions. Therefore we uncoupled flow and volume by linearizing expiratory flow (flow-controlled expiration). This enabled calculation of compliance separately for inspiration and expiration. We hypothesized that the volume-dependent intratidal compliance profiles differ between inspiration and expiration, describing a dynamic hysteresis behavior.

## Methods

In five Swedish Landrace Hybrid pigs weighing 26 ± 2 kg the lungs were ventilated in the volume-controlled mode. PEEP was set to 0, 6, 12 and 15 cmH_2_O. The flow-controlled expiration was realized by a computer-controlled expiratory resistance which was adjusted in a fashion that expiratory flow was strongly limited in the beginning and continuously facilitated towards the end of expiration. Using the gliding-SLICE method [1], intratidal inspiratory and expiratory compliance profiles were calculated from inspiration data only and from expiration data only, respectively. The dynamic hysteresis area was calculated as the area within the dynamic tracheal pressure-volume loop. The relative hysteresis area was calculated as the quotient of hysteresis area divided by the rectangular area which is limited by the minima and maxima of pressure and volume of the respective pressure-volume loop [2].

## Results

Intratidal compliance profiles of inspiration and expiration differed strongly in mean value and slope at low PEEP. With increasing PEEP the inspiratory compliance profile approximated closer to the expiratory compliance profile. This was accompanied by a decreased relative hysteresis area by 26%.

## Conclusion

Flow-controlled expiration allows for calculation of respiratory system compliance separately for inspiration and expiration. This compliance displays the hysteresis behavior of the respiratory system during uninterrupted ventilation. Such analysis, which is similar to the time-honored quasi-static hysteresis area analysis, could be helpful for finding an optimal PEEP.
